# Guidelines for Clinicians and Pathologists on Performing Skin Biopsies and Reporting on Suspected Cutaneous Squamous Cell Carcinoma

**DOI:** 10.3390/curroncol32120689

**Published:** 2025-12-04

**Authors:** May Chergui, Margaret Redpath, Chang Shu Wang, Alex Mlynarek, Khashayar Esfahani, Stephanie Thibaudeau, Khalil Sultanem, Joël Claveau

**Affiliations:** 1 Department of Pathology, McGill University Health Centre, Montreal, QC H4A 3J1, Canada; 2 Department of Pathology, Sir Mortimer B. Davis Jewish General Hospital, McGill University, Montreal, QC H3T 1E2, Canada; 3 Department of Nuclear Medicine and Radiobiology, Centre Hospitalier Universitaire de Sherbrooke, Université de Sherbrooke, Sherbrooke, QC J1H 5H3, Canada; 4 Department of Otolaryngology Head and Neck Surgery, Sir Mortimer B. Davis Jewish General Hospital, McGill University, Montreal, QC H3T 1E2, Canada; 5 Division of Oncology, Department of Medicine, Sir Mortimer B. Davis Jewish General Hospital, McGill University, Montreal, QC H3T 1E2, Canada; 6 Division of Plastic Surgery, Department of Surgery, Montreal General Hospital, McGill University, Montreal, QC H4A 0B1, Canada; 7 Division of Radiation Oncology, Department of Oncology, Sir Mortimer B. Davis Jewish General Hospital, McGill University, Montreal, QC H3T 1E2, Canada; 8 Division of Dermatology, Department of Medicine, Melanoma and Skin Cancer Clinic, Centre Hospitalier Universitaire de Québec, CRCEO, Université Laval, Quebec City, QC G1R 3S1, Canada

**Keywords:** cutaneous squamous cell carcinoma, biopsy, dermatopathology reports, pathology

## Abstract

Cutaneous squamous cell carcinoma (CSCC) is the second most common skin cancer after basal cell carcinoma. When squamous cell carcinomas in situ are included, nonmelanoma skin cancer incidence is nearly equal between CSCC and basal cell carcinoma. The incidence of CSCC has been increasing worldwide in recent decades, and despite the effectiveness of office-based therapies, patients with high-risk lesions associated with advanced CSCC face high rates of recurrence and mortality. This underscores the importance of accurate diagnoses and clear criteria to define high-risk lesions for prognosis and better treatment strategies. However, variability exists in CSCC registration practices internationally, and differences in pathology reporting likely contribute to an underestimate of the true burden of disease. Thus, there is a need to refine elements included in skin biopsy reports to provide a precise representation of the high-risk features of CSCC to improve patient care. In this review, a multidisciplinary group of Canadian experts discuss clinical considerations and provide key guidance and practical strategies surrounding skin biopsy techniques, completion of requisition forms, and dermatopathology reports for CSCC. This article summarizes the expert panel’s recommendations with the goal of improving diagnosis and pathological reporting of biopsy specimens to achieve better patient outcomes for CSCC.

## Simple Summary

A common type of skin cancer, called squamous cell carcinoma, is becoming more widespread, and for patients with more serious forms, it can be life-threatening. The way skin samples are currently taken, the information is sent to the lab, and how lab reports are written often differ, making it difficult to get accurate diagnoses and the most effective treatment. To address this, a group of Canadian medical experts has developed clear guidelines. They recommend better methods for doctors to take skin biopsies, more complete information to include on lab request forms, and standardized lab reports that highlight crucial risk factors. Following these recommendations will lead to more precise diagnoses and help medical teams make quicker, more appropriate treatment decisions, ultimately improving patient health and reducing the chances of serious complications from this skin cancer.

## 1. Introduction

Cutaneous squamous cell carcinoma (CSCC) is the second most common skin cancer, with increasing global prevalence in recent decades [1,2,3]. Unsurprisingly, the incidence of CSCC is projected to continue increasing due to the growing elderly population and cumulative lifetime sun exposure [4,5,6]. In fact, as a major risk factor, sun exposure contributes to the high incidence of CSCC arising from the head and neck, including the ear, cheek, lip, and scalp [7,8]. Fortunately, most cases of CSCC are associated with favourable outcomes and excellent prognosis. However, patients presenting with high-risk tumours associated with advanced or metastatic CSCC face high rates of recurrence and mortality [9,10]. Ultimately, the increasing incidence and poor survival rates for patients presenting with aggressive CSCC highlight the importance of having clear diagnoses and accurately defining high-risk lesions to better inform therapeutic strategies.

To refine the diagnostic accuracy of biopsy specimens for improved patient care, limitations in current clinical practices must be addressed. For example, the absence of standardized protocols for CSCC biopsy methods and insufficient clinical information provided on requisition forms can significantly delay diagnosis and/or lead to misdiagnosis [11,12,13,14]. Additionally, histopathologic reporting in CSCC is not uniform, and many features of prognostic value are lacking or go unreported [15]. Ultimately, effective communication between the pathologist and the clinician is imperative to establish the most accurate diagnosis and stage the patient, thereby permitting an appropriate management plan to be initiated as rapidly as possible. Breakdowns within the skin biopsy diagnostic process can substantially influence the quality of dermatopathological interpretation, which ultimately impacts the quality and safety of patient care, as well as healthcare utilization and costs [11,15,16,17].

In an ideal scenario, the requesting clinician would provide an adequate biopsy specimen and sufficient relevant clinical information within a requisition form to the dermatopathologist to yield the most accurate diagnosis and help identify high-risk CSCC features. In turn, the dermatopathologist would perform a histopathologic interpretation and generate a comprehensive pathology report that highlights the key prognostic features to help guide physicians in the management of CSCC. The objective of this review is to provide best practices for clinicians and dermatopathologists to improve skin biopsy processes, requisition form completion, and dermatopathology reporting in CSCC with the goal of improving diagnostic accuracy of biopsy specimens for better patient care.

## 2. Key Recommendations for Skin Biopsy Procurement in CSCC

A biopsy is essential to confirm the clinical diagnosis and to identify histopathologic parameters for risk stratification which will guide patient management [9,18]. These goals are only possible with an adequate biopsy specimen. The selection of appropriate tissue sample requires important considerations, such as anatomic location and tissue characteristics, whether the tissue is sampled partially (incisionally) or completely (excisionally), and an understanding of the suitability of various biopsy techniques [19,20,21]. Key characteristics of incisional and excisional biopsies are summarized in Table 1, and an overview of common skin biopsy techniques and their clinical importance is outlined in Table 2.

**Table 1:** Key characteristics of incisional and excisional biopsy removal strategies [12,13,19,20,21,22,23].

Biopsy Strategy	Incisional	Excisional
**Description**	Partial or incomplete sampling of a lesion	Complete removal of a lesion with margins of 1–5 mm of normal skin
**Advantages**	Useful when skin architecture needs to be maintained May be used when aesthetics and avoidance of disfigurement are desired May be used for large necrotic tumours	Offers diagnostic and potential therapeutic benefit Allows for clear diagnosis and appropriate staging Confers therapeutic potential that may be curative in some cases May be used when tumour removal is required as quickly and completely as possible
**Disadvantages**	Potential for sampling errors and issues Theoretical risk for opening the tumour capsule and dissemination No therapeutic benefits	Longer procedure requiring higher level of skills and resources May be limited by size of defect and tumour proximity to important anatomic areas or structures

**Table 2:** Overview of common skin biopsy techniques used in CSCC [12,13,19,20,21,22,23,24].

Biopsy Strategy	*Incisional (Partial Sampling of Lesion) or Excisional (Complete Removal of Lesion)*				
Biopsy Type	Punch ○ Incisional or excisional	Shave ○ Incisional or excisional	Saucerization ○ Incisional or excisional	Wedge ○ Incisional	Ellipse ○ Excisional
**Technique Description**	Specimen is punched out using a disposable or sterilizable punch	Specimen is shaved off using a flexible, gripped blade	Specimen is removed with a curved blade, yielding a thick disk of tissue	Specimen is removed through a triangular-shaped stab incision with a scalpel blade, yielding a cone of tissue	Specimen is removed with a scalpel blade, yielding an elliptical piece of tissue
**Ease of Performance**	++++	+++	+++		+
**Time-/Resource- Effective**	++++	+++	+++		+
**Minimally Invasive**	++++	++++	+++		+
**Yield/Tissue Adequacy for Diagnosis**	++	+	+++		++++
**Yield/Tissue Adequacy for Staging**	+	+	++ (incisional)		++++
			++++ (excisional saucerization)		
**Clinical Relevance**	Useful for general skin cancer diagnosis Allows better visualization of depth	Useful for general skin cancer diagnosis Generates broad sample that can aid diagnosis of the primary site as well as precursor lesions	Useful when accurate diagnosis and staging information is required Saucerization allows possible resection of whole tumour		Useful when accurate diagnosis and staging information is required Allows resection of whole tumour and may be therapeutic
**Clinical Use**	May be used for large tumours involving the face	Not ideal for CSCC	May be used for small lesions on the trunk of the body		May be used for deep or large lesions
**Other**	Due to small sample size, may not provide representative staging information	Often not deep enough to capture full tumour thickness required for staging; thus, other methods are preferred	Not always possible in more delicate areas May leave larger areas to be resected		Not always possible in more delicate areas Requires training and surgical expertise

++++ and green colour denote very favourable characteristics; +++ and light green colour denote favourable characteristics; ++ and light orange colour denote less favourable characteristics; + and orange colour denote unfavourable characteristics. CSCC: cutaneous squamous cell carcinoma.

### 2.1. Biopsy Considerations

The main distinction between both biopsy types is the surgical intent of the procedure: an incisional biopsy removes a *portion* of the lesion, whereas an excisional biopsy removes the *entire* lesion [12,22,23]. Clinicians should always use their judgement when choosing the optimal biopsy technique for their clinical scenario, considering the advantages and disadvantages of each type. On the one hand, incisional biopsies are characterized by greater ease of performance, are usually more time-effective, and are potentially less invasive compared to their excisional counterparts. However, incisional biopsies may provide incomplete diagnostic information. In fact, they may not be representative of the entire tumour and may not capture the lesion architecture or aggressive features.

On the other hand, excisional biopsies provide high tissue yield for diagnosis, offer more tumoural information for staging purposes, and confer potential therapeutic benefit.

Nevertheless, excisional biopsies are not always possible because some lesions may be too large, arise on cosmetically sensitive areas, and/or areas that may have a healing or functional impact on the patient. In these cases, an incisional biopsy may be favoured to reduce the impact of the procedure on the patient, while still providing enough histopathologic information about the disease to guide management. Regarding incisional biopsies, dermoscopy can be used to guide partial biopsies and help select the best area to sample. Dermoscopy is a noninvasive technique that enables in vivo evaluation of suspicious skin lesions and visualization of subsurface skin structures [25,26]. Dermoscopy has been shown to increase the sensitivity of CSCC diagnosis by revealing distinct patterns associated with various lesion types and stages of progression (e.g., SCC in situ/Bowen’s disease vs. invasive SCC) [27,28]. Specific dermoscopic features can serve as indicators for guiding targeted biopsies, enabling clinicians to precisely identify areas within a lesion most suspicious for malignant transformation or invasion, thereby optimizing biopsy yield and diagnostic accuracy (e.g., glomerular/punctiform vessels in in situ SCC vs. keratin masses and polymorphic vessels in invasive SCC) [27,28].

In preparation for the biopsy, the exact site should be noted with a surgical marker, as it may become obscured after injection with the local anaesthetic. Determining which part of a lesion to biopsy requires a balance between choosing a representative sample and avoiding areas with secondary changes (e.g., regeneration, scarring, ulcers, necrosis, recent treatment) which may pose interpretative challenges, as they may obscure the underlying pathology. In large and/or polymorphic lesions, multiple biopsies should be performed to ensure appropriate representation of the tumour’s heterogeneity [21,22,23,29].

### 2.2. Biopsy Techniques

In parallel to the aforementioned considerations, there are several biopsy techniques to consider, each having their own advantages and yielding disadvantages, as outlined in Table 2. Generally, techniques with the greatest ease of performance are generally more time- and resource-effective, require less surgical expertise, and are less invasive, resulting in more cosmetically favourable results. However, these simpler biopsy techniques are associated with many limitations, such as low tissue yield that can hinder the evaluation of features required for diagnostic staging, and are of minimal therapeutic benefit. In contrast, techniques that allow for more extensive sampling have numerous advantages, including higher tissue yield, improved diagnosis and staging, and have potential therapeutic benefit, but are more challenging to perform.

Notably, superficial biopsy techniques should be avoided as they may yield specimens lacking even, full-thickness epidermis (e.g., shaves and curettages) and often leave the lateral edges of lesions unsampled (e.g., punch) [13,30]. By definition, if the biopsy is not deep enough to sample the dermis, then a diagnosis of invasive carcinoma cannot be made [31].

Curettage is commonly used as a therapeutic option in practice, but it should be avoided as a diagnostic procedure. This technique removes a superficial specimen using an ablative method with a curette, typically in conjunction with cautery. Although curettage confers some therapeutic benefit when performed on small lesions, it is generally advised against as a first diagnostic procedure due to its poor tissue yield and inability for diagnostic staging [12,19]. Nowadays, when this technique is used as a therapeutic option, it is performed in conjunction with a saucerization and electrodessication, or cryosurgery [32].

### 2.3. Biopsy Handling and Storage

Upon sample retrieval, the biopsy specimen should be rapidly placed in formalin and undergo minimal fluctuations in temperature [19,22]. It is critical to ensure that the specimen is fully immersed in the formalin solution [19]. Additional precautions should be made to avoid cauterizing the sample, because this can obscure the cytology of the cells and make it difficult to diagnose and/or assess the margin status. Squeezing or crushing the biopsy specimen with forceps should also be avoided, as this may give rise to a compression artifact after processing [12,19,21].

### 2.4. Summary on Key Recommendations for Skin Biopsy Processes in CSCC

Despite its seemingly simplistic nature, the skin biopsy is an essential clinical skill of any clinician. If performed correctly, it can be completed rapidly and comfortably for the patient, and yield significant diagnostic information [19,21,23]. If performed incorrectly, it can lead to delays in diagnosis and treatment and even result in inappropriate management of the patient. Ultimately, accurate diagnosis of CSCC begins with the selection of an appropriate biopsy technique and the implementation of an effective preservation strategy [19,21,23]. A summary of key recommendations for the different skin biopsy processes in CSCC is reported in Table 3.

**Table 3:** Summary of key recommendations to improve skin biopsy processes [12,21,22,23,29,30,31].

Consensus Recommendations	Implications
**Choosing optimal biopsy site**	
Choose sites with well-formed established lesions Avoid areas with secondary changes that may obscure underlying pathology (e.g., regeneration, scarring, ulcers, necrosis, recent treatment) May depend on site availability for biopsy May involve consideration of cosmetic, healing, and/or functional impact on patient	Yields specimen most representative of the lesion while avoiding tumour sampling issues as much as possible Captures invasive and/or aggressive tumour growth patterns that may be present Ensures better pathological interpretation of the biopsy specimen to inform treatment plans
**Performing appropriate biopsy technique**	
Select biopsy strategy (i.e., incisional or excisional) prior to definite treatment Excisional biopsies allow clear diagnosis with the potential for therapeutic benefit There is no single optimal biopsy technique May depend on tumour size, location, morphology May depend on the purpose of the biopsy (partial sampling/scouting vs. extensive tissue evaluation) and treatment approach	Enables accurate assessment of key pathology elements (i.e., margin status, depth of invasion) when the appropriate technique is chosen Ensures better pathological interpretation of biopsy specimen to guide treatment plans Minimizes discomfort, trauma, risk for wound infection, scar, or loss of function for patients
**Obtaining adequate tissue sample**	
Sample enough tissue to capture breadth and/or depth of the lesion May involve obtaining adjacent normal tissue for comparative analyses May require larger specimens if margin assessment is required	Provides the pathologist sufficient tissue that fully displays lesion architecture Captures invasive and/or aggressive tumour growth patterns that may be present Yields a high level of clinical information that enables accurate diagnosis and assessment of key pathology elements to guide therapeutic plan
**Proper handling of biopsy specimen**	
Store specimen in optimal fixative/transport medium Fully immerse specimen Handle specimen as minimally as possible Avoid temperature variations Avoid cauterization	Maintains specimen tissue integrity and avoids damage Ensures better histopathological interpretation of the biopsy specimen and accurate diagnosis by avoiding tissue artifacts

## 3. Key Recommendations for Requisition Forms in CSCC

Requisition forms that accompany biopsy specimens are a critical link between the requesting clinician and the pathologist. Clinical information necessary for histopathologic interpretation may be easily misinterpreted or lost without effective communication [11,14,16,17,33,34]. To ensure that knowledge is being transferred as completely and accurately as possible, requisition forms must be optimized to convey essential patient and lesion information, as well as processing directives for the pathologist. An outline of the proposed key information and features to be captured in an ideal requisition form is illustrated in Table 4 (a proposed form is available in the Supplementary Materials, Figure S1).

**Table 4:** Proposed CSCC requisition form criteria based on consensus recommendations [1,2,9,17,19,21,23,29,31,35,36,37].

Category	Items to Include
Patient Information	Age Sex Immunosuppression status Relevant personal medical history Previous procedures/therapies to the current lesion Other patient information
Pathology Processing Directives	Biopsy method used and purpose (incisional [punch, shave, wedge, saucerization]; excisional [punch, shave, saucerization, ellipse]) Re-excision (if yes, indicate previous biopsy result) Expedited review (if yes, indicate planned procedure/next steps) Margin assessment Other information
Lesion Information	Lesion width/largest diameter New or recurrent lesion Chronic features (wounds, ulcers, burns, other) Suspected nodal involvement Precise anatomic location of biopsy (body schema) and/or measurement from anatomic landmarks Clinical photos/images Other lesion information

### 3.1. Patient Information

Key elements of a patient’s medical history or status should be noted, including previous procedures or treatments to the current lesion, as this can significantly alter the lesion’s morphology [9,19]. Information that should be reported in the requisition form includes a previous history of hematological malignancies (e.g., chronic leukemia and lymphoma), as well as other factors associated with immunosuppression, such as an organ transplant. These details are important to convey because they provide clinical context that assists in the interpretation of the histologic features, and enables the pathologist to comment on prognostically relevant factors that may impact risk stratification [2,9,29]. Of note, immunosuppressed patients can develop CSCC with a deceptively bland histologic appearance, so providing this clinical context prompts pathologists to lower their threshold for determining malignancy [31,38].

Additional relevant clinical context which should be provided on the dermatopathology requisition includes predisposing conditions that are associated with a higher risk of CSCC, including extensive presence of actinic keratoses and chronic inflammatory dermatoses such as hidradenitis suppurativa, chronic ulcers, or lichen sclerosus [39,40,41]. Supplying this information facilitates accurate histopathologic interpretation and risk stratification.

### 3.2. Pathology Processing Directives

Processing directives must be provided to the pathologist. Importantly, the chosen biopsy technique and its intended purpose must be indicated.

The clinician must specify whether the review should be expedited for advanced cases of CSCC or for patients who are elderly, immunosuppressed, and/or have other high-risk features. Requests should also be prioritized for those with advanced cases involving areas that may have functional impact (i.e., head, neck, hands, feet), or in cases where nodal or distant metastases are suspected. Additionally, patients with significant/multiple comorbidities, pigmented lesions, or who are booked for immediate treatment (e.g., Mohs micrographic surgery) should be candidates for an expedited pathology report [2,31]. In such cases, the purpose/rationale and directives for expedited review should be captured in the requisition form to ensure those specimens are prioritized during triage for an expedited analysis and review.

Additionally, the requesting clinician should communicate when margin evaluation is required for excisional specimens so that margins can be assessed accordingly [23,31]. For example, if pathologists are informed that the biopsy or excision is being performed with curative intent, they will report the margin status to the clinician; or for poorly differentiated lesions, if radiation is being considered, they will assess the margins in a timely manner and measure the precise distance [31,35].

### 3.3. Lesion Information

Indicating the type of biopsy on the request to the pathologist, and specifying whether the specimen is incisional and diagnostic or excisional and therapeutic should be routine practice [23,31]. Maximum clinical diameter of the lesion is the main criterion used to determine the pathologic stage of the primary tumour and thus should be specified whenever possible. This is particularly important when the biopsy specimen is small, as is the case with punch or incisional techniques. Moreover, any disease or lesion recurrence should be noted, as it may affect histopathologic interpretation and subsequent risk stratification [9,31].

The anatomical site of carcinoma specimens is often noted by clinicians, but the lack of detail can often be problematic, especially if a second excision is needed. Ideally, two precise anatomical landmarks (e.g., labial commissure, ala nasi, tragus) should be provided as points of reference to communicate lesion location. An additional easy solution to this is the use of a simple body schema, as illustrated in Figure S2, to indicate the precise location of lesions for pathologists and clinicians [1,31,36]. If a re-excision is required in the future for residual or recurrent disease, this will help guide the clinician, especially if there are multiple lesions in one anatomical location, or in cases where it is not the same clinician who performed the original biopsy. Additionally, in cases where there may be multiple biopsy sites, knowing the precise location of, for example, two biopsies relative to each other is also invaluable information for the pathologist, as this can help them understand whether they are assessing two lesions or two areas of the same lesion [1,23,42,43,44].

Clinical photographs of the lesion may provide visual context for the pathologist to aid with diagnosis and localization [17,36,37]. However, it is associated with administrative barriers and may only be possible when clinicians have direct access and lines of communication with the pathologist. Widespread adoption of more specific electronic medical records will help facilitate this task [36,45].

Providing the pathologist pertinent clinical information can help avoid the over- or underdiagnosis of lesions. For example, if a patient is repetitively scratching a lesion, this can lead to inflammation, reactive keratinocyte atypia, and pseudoepitheliomatous hyperplasia that can be easily mistaken for carcinoma [38,46]. Similarly, chronic ulcers in patients with venous stasis also tend to develop pseudoepitheliomatous hyperplasia; thus, informing the pathologist that they are assessing a chronic wound will prompt them to increase their threshold for determining changes that should be considered malignant [47,48]. Another pitfall for overcalling a lesion is thermal injury, as burn lesions can display cytologic atypia and significant mitotic activity during the repair process [49]. Previous biopsy results, when available, should be shared to provide important clinical context, which may influence the extent to which a pathologist works up a case [19,21].

### 3.4. Summary on Key Recommendations for Requisition Forms in CSCC

Ultimately, effective communication between clinicians and pathologists is critical for an accurate pathologic interpretation, which will directly impact patient care [16,50]. Table 5 provides a summary of key recommendations to improve CSCC requisition forms and facilitate this interaction. To further improve patient outcomes, clinicians may consider increasing their understanding of general pathology through electives, tumour boards, or by working closely with a pathologist [51]. This will consequently lead to enhanced clinicopathologic communication and correlation, and thus, more accurate diagnoses and better patient management.

**Table 5:** Summary of key recommendations to improve CSCC requisition forms [2,9,16,29,31,34,52].

Consensus Recommendations	Implications
**Providing sufficiently detailed patient information**	
Provide key elements of the patient demographics, clinical presentation, and medical history Provide relevant information on any potential risk factors (e.g., immunosuppression, organ transplant status)	Provides clinical context that can greatly assist in interpretation of the histologic findings Ensures better pathological interpretation of the biopsy specimen to inform treatment plans Enables the pathologist to comment on prognostically relevant factors that may impact risk stratification
**Providing sufficiently detailed lesion information**	
Specify anatomic location of the tumour and recurrent lesion status Specify clinical size/largest diameter of lesion, which is needed for tumour staging Record any unusual features that may be diagnostically important (e.g., chronic wound, thermal injury) May involve providing a clinical photograph to accompany the biopsy specimen, if appropriate, to supplement clinical information	Provides clinical context that can greatly assist in interpretation of the histologic findings Ensures better pathological interpretation of the biopsy specimen to inform treatment plans Enables the pathologist to comment on prognostically relevant factors that may impact risk stratification
**Indicating whether biopsy specimen comprised a portion of the lesion or the entire lesion**	
Specify whether biopsy is incisional (lesion sampling) or excisional (lesion removal)	Provides clinical context that can help pathologists determine which analyses should be conducted Ensures better pathological interpretation of the biopsy specimen to inform treatment plans Helps avoid diagnostic pitfalls related to tissue sampling and tumour heterogeneity
**Indicating purpose of request**	
Specify whether the biopsy purpose is diagnostic (to determine and/or confirm diagnosis) or therapeutic/curative (complete excision with clear margins)	Provides clinical context that can help pathologists determine which analyses should be conducted Enables the pathologist to comment on prognostically relevant factors that may impact therapy
**Clearly indicating when margin assessment is required**	
Ensure biopsy specimens are appropriately oriented when margin status is required May be necessary for future procedures/treatments	Ensures that biopsy samples are prepared and inked accordingly so that margins may be assessed Ensures better pathological interpretation of the biopsy specimen to inform treatment plans
**Requesting expedited review for elderly and/or high-risk patients**	
Prioritize requests for those with: ○ Advanced cases involving areas that may have functional impact (i.e., head, neck, hands, feet) ○ Hematologic malignancies ○ Organ transplant ○ Immunosuppression ○ Final treatment not yet initiated (e.g., Mohs surgery, radiation) ○ Significant/multiple comorbidities ○ Pigmented lesions	Ensures that specimens are triaged and prioritized accordingly for pathological analysis and reporting Enables the pathologist to comment on prognostically relevant factors that may impact therapy Allows crucial treatment decisions to be made in a timely manner when diagnoses are established more quickly

## 4. Key Recommendations for Dermatopathology Reporting in CSCC

The main purpose of the pathology report is to provide the clinician with an accurate diagnosis of CSCC with as much prognostic information as possible [21,29]. The meticulous recording of pathological parameters in the dermatopathology report is important because key histological parameters play a significant role in defining individual patient treatment plans [31,52]. The sections outlined in Table 6 (a proposed form is available in the Supplementary Materials, Figure S3) provide diagnostic context that inform prognosis and risk stratification to enable appropriately tailored treatment decisions for patients with CSCC.

**Table 6:** Proposed CSCC pathology report content based on consensus recommendations [31,52].

Category
Histologic subtype Degree of differentiation Level of invasion Maximum tumour thickness Perineural invasion/lymphovascular invasion Margin status assessment Nodal disease Aggressive histologic characteristics Number of high-risk features Additional comments

### 4.1. High-Risk Criteria

Key prognostic features that should be highlighted in a pathology report for risk stratification in CSCC include the degree of differentiation, level, and depth of invasion, perineural invasion (PNI), and the presence of high-risk histological subtypes [2,29,53,54]. Furthermore, lymphovascular invasion (LVI), single-cell spread, and poor/sarcomatoid differentiation have been reported as important elements associated with poor prognosis, but have not been definitively shown to be independent risk factors [2]. LVI specifically has been linked to metastatic spread [55]. Relevant stromal features include desmoplasia, which inform aggressiveness and invasion risk [56].

#### 4.1.1. Tumour Subtype and Degree of Differentiation

Performing histology on a skin biopsy specimen yields a wealth of information that must be noted in an CSCC pathology report. Particularly, histologic subtype must be reported because certain subtypes, such as acantholytic, adenosquamous, and spindle cell, are considered high-risk variants, whereas verrucous and keratoacanthoma are considered low-risk clinical variants of SCC [2,29,38].

The grade or degree of differentiation of the tumour is an important feature that must be included in CSCC pathology reports because it is correlated with prognosis/patient outcome and may affect the therapeutic plan [9,31]. Of note, desmoplastic subtype is an important factor to be considered for an adjuvant radiotherapy indication [29,57]. Additionally, poorly differentiated tumours may require further analyses. For example, if the tumour cells are so poorly differentiated and no longer resemble keratinocytes, then immunohistochemical stains may be required to confirm that the tumour is a squamous cell carcinoma [6,21,31].

#### 4.1.2. Level of Invasion and Tumour Thickness

Other important criteria within a CSCC pathology report are the level of invasion and tumour thickness. Firstly, it should be specified whether the tumour is in situ, invading the dermis, extending through the subcutaneous fat, or invading the bone. Notably, invasion occurring beyond the subcutaneous fat is considered a high-risk feature. Furthermore, the presence of PNI and/or LVI should be specified as they are high-risk features. If PNI is present, it should be documented whether the diameter of the largest affected nerve is <0.1 mm or ≥0.1 mm, because risk of nodal metastasis is significantly higher in patients with large-calibre versus small-calibre PNl [2,9,29,31].

Tumor thickness in CSCC can be measured from the granular layer of adjacent normal epidermis to either the base of the tumor or the tissue plane of deepest invasion [58]. If the lesion is ulcerated, or an epithelial defect is present, the measurement starts from the base of the ulceration/defect, extending down to the deepest identifiable invasive tumor cells [52]. However, care must be taken to clearly distinguish between “tumor thickness” and “depth of invasion,” as they are not necessarily the same. In exophytic tumors, for example, the “tumor thickness” will be great, however the “depth of invasion” is not necessarily deep. The maximum tumour thickness should be specified to the nearest millimetre, as dimensions may provide insights on risk severity. For instance, if the thickness is ≤2 mm, the tumour is not generally associated with metastatic potential and is amenable to complete curative excision. Conversely, a thickness of >6 mm represents a high-risk feature while a thickness of >10 mm is associated with high mortality potential [31,52,59].

#### 4.1.3. Tumour Margins and Nodal Disease

Margin assessment is an essential element of prognostic information [29,44,52]. Margin status is an especially important factor influencing management for radiation oncologists. However, reporting of margin status is not standardized. Furthermore, histopathologic measurements of margins do not necessarily correlate with degree of margin clearance clinically because of technical variables such as tissue shrinkage during fixation [6,60,61,62,63]. Although pathologists often use their best judgement to determine when margin assessment is necessary, it is best practice for clinicians to provide clear directives when margin status is needed. Margin assessment typically involves specifying whether the margins are involved by in situ or invasive carcinoma, the extent of disease present at the margin (focal or extensive), and which margins are involved (peripheral or deep). Moreover, any aggressive features that extend close to the margins should be reported [29,31].

Another important piece of information that may impact therapeutic strategy is whether the patient has nodal disease. When reporting on nodal disease, the total number of lymph nodes present in the specimen, the number of lymph nodes involved by metastatic carcinoma, and the size of the tumour deposits should be noted [52,64,65]. Furthermore, the presence of extranodal extensions should be specified, as this may be associated with worse prognosis [8,52,66].

#### 4.1.4. Other Important Reporting Criteria

Additional factors should be mentioned in an CSCC dermatopathology report if present and/or applicable, including any aggressive histological characteristics and the number of high-risk features [29]. For example, desmoplasia and sarcomatoid differentiation are considered high-risk features and should be reported, as this may impact the therapeutic plan [2,9].

Ultimately, in conjunction with clinical and radiologic criteria/findings reporting on these histologic criteria provides diagnostic context that informs staging, prognosis, and risk stratification to best tailor treatment (primary, neo-adjuvant and adjuvant) and management decisions for patients with CSCC [57,67,68].

### 4.2. Synoptic Reporting

Typical pathology reports are free text and therefore can be prone to omission of key details and inconsistencies in formatting. The need for more comprehensive reporting in tumour pathology has led to synoptic reporting, a process for reporting specific and necessary data elements in a structured and systematic format [69,70]. The synoptic reporting method for preparing and submitting healthcare reports, which incorporates evidence-based best practices and validated data elements, has proven beneficial for cases of cutaneous melanoma in comparison to nonsynoptic reports in terms of completeness of data capture [70,71]. As such, synoptic reporting practices adopted in melanoma may be leveraged in CSCC. Notably, synoptic reporting can be greatly beneficial in high-risk CSCC cases, as it standardizes the report for ease of interpretation [72]. The College of American Pathologists (CAP) recently published updated Protocols for the Examination of Specimens from Patients with Cutaneous Squamous Cell Carcinoma of the Head and Neck, wherein synoptic reporting is described [52]. Use of synoptic reporting may be reserved for more advanced cases (e.g., poorly differentiated, basosquamous, immunosuppressed) and should be clearly indicated by the requesting clinician in the requisition form if there is a preference for this method.

### 4.3. Summary on Key Recommendations for Dermatopathology Reporting in CSCC

As histology is the gold-standard requirement for the diagnosis of CSCC, histopathological reporting is critical and requires a standardized pathology report to ensure key criteria and aggressive growth patterns, if present, are captured in order to guide appropriate therapy [18]. A summary of key recommendations to improve CSCC pathology reports is illustrated in Table 7.

**Table 7:** Summary of key recommendations to improve CSCC pathology reports [2,9,29,31].

Consensus Recommendations	Implications
**Reporting histologic subtype**	
Specify the subtype variant/features ○ Acantholytic, adenosquamous, and spindle cell subtypes are considered high-risk features ○ Verrucous and keratoacanthoma subtypes are considered prognostically favourable features	Provides diagnostic context that informs prognosis and risk stratification so that appropriately tailored treatment decisions can be made Provides information on potential risk of local recurrence and metastasis that may affect therapeutic plan
**Reporting degree of tumour differentiation/grade**	
Specify whether the tumour is well, moderately, or poorly differentiated ○ Poor differentiation is a high-risk feature Further immunohistochemistry analyses may be needed for poorly differentiated tumours	Provides diagnostic context that informs prognosis and risk stratification so that appropriately tailored treatment decisions can be made Provides information on potential aggressive growth pattern that may affect therapeutic plan
**Reporting level of invasion**	
Specify whether the tumour is in situ, in the dermis or subcutaneous tissue, beyond subcutaneous fat, or deeper at the bone ○ Invasion beyond subcutaneous fat is a high-risk feature Specify presence or absence of perineural (focal/extensive) and/or lymphovascular invasion ○ Presence of any perineural or lymphovascular invasion is a high-risk feature Specify diameter of the largest affected nerve (when ≥0.1 mm) if perineural invasion is present	Provides diagnostic context that informs staging and risk stratification so that appropriately tailored treatment decisions can be made Provides information on potential risk of local recurrence and metastasis that may affect therapeutic plan
**Reporting maximum tumour thickness**	
Specify thickness to the nearest millimeter ○ ≤2 mm is not generally associated with significant metastatic potential and is amenable to complete curative excision ○ >6 mm is a high-risk feature and >10 mm is associated with high mortality potential	Provides diagnostic context that informs staging and risk stratification so that appropriately tailored treatment decisions can be made Provides information on potential risk of local recurrence and metastasis that may affect therapeutic plan
**Reporting margin status when requested and/or necessary**	
Specify whether the margin is involved by in situ carcinoma or invasive, the extent of the disease present at the margin (focal or extensive), and which margin is involved: ○ Peripheral margins ○ Deep margins Report any aggressive features extending close to margins	Provides information on completeness/adequacy of primary tumour excision and clearance of margins for biopsies with therapeutic intent Provides information on potential risk of recurrence that may affect therapeutic plan Ensures the multidisciplinary team are better able to plan therapy, should additional surgery and/or radiation therapy be required
**Reporting nodal disease**	
Specify whether lymph nodes are present in specimen and number present/involved Specify whether extranodal extensions are present ○ Presence of extranodal extensions is associated with worse prognosis	Provides information on the tumour burden Provides diagnostic context that informs staging and risk stratification so that appropriately tailored treatment decisions can be made Provides information on potential risk of regional and/or distant metastasis that may affect therapeutic plan Ensures the multidisciplinary team are better able to plan therapy, should surgery, radiation therapy, and/or systemic therapy be required
**Commenting on other factors if applicable**	
Aggressive histologic characteristics ○ Presence of desmoplasia and sarcomatoid differentiation are considered high-risk features Number of high-risk features	Provides additional clinically relevant/useful information that may impact therapeutic plan Provides diagnostic context that informs staging, prognosis, and risk stratification so that appropriately tailored treatment decisions can be made

## 5. Conclusions

**Figure 1:**
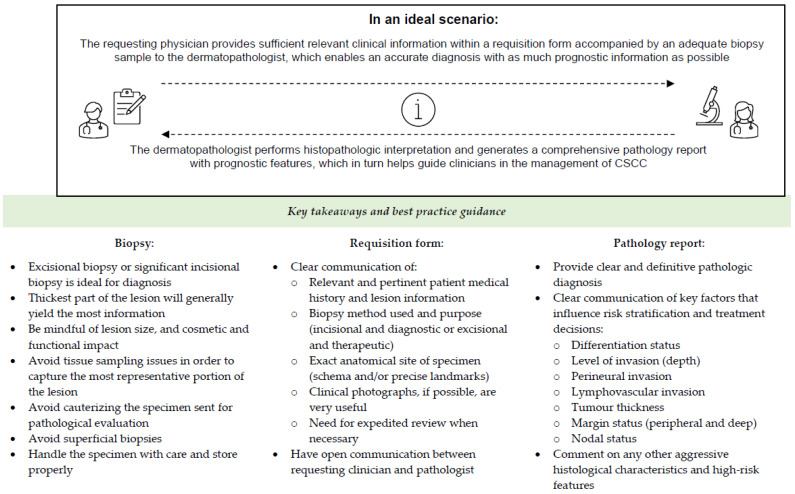
Key takeaways for improving skin biopsy processes and clinician–pathologist communication through requisition forms and pathology reports.

Cases of CSCC have been increasing, and although most cases are associated with favourable outcomes, for patients with high-risk features, local recurrence and metastatic disease can significantly increase risk of mortality. To provide improved patient care, the limitations of current clinical practices in CSCC must be addressed. Currently, biopsy specimens are too often inadequate, and clinical information provided on the skin biopsy requisition form is often incomplete or inaccurate, which can greatly hinder the pathologists’ abilities to efficiently make definitive diagnoses. Furthermore, histopathologic reporting of CSCC is not uniform, and if comments on key high-risk features are not captured, this can have major therapeutic implications. Given the multidisciplinary nature of current oncologic treatment, it is not surprising that various healthcare professionals rely on different types of information; thus, there is a need for more complete and standardized reporting in oncologic pathology. The key takeaways for improving skin biopsy processes and clinician–pathologist communication through requisition forms and pathology reports are outlined in Figure 1. Ultimately, the information contained within this review is intended to shed light on the existing limitations of CSCC diagnosis and subsequent management planning and offer expert-based consensus recommendations for standardized clinical practices to improve patient care.

## Data Availability

Data sharing not applicable—no new data generated.

## Supplementary Materials

The following supporting information can be downloaded at: https://www.mdpi.com/article/10.3390/curroncol32120689/s1. Figure S1: Proposed cSCC requisition form based on consensus recommendations. Figure S2: Example of a body schema that can be helpful for the requesting clinician to communicate to the pathologist where skin biopsy samples have been taken. Figure S3: Proposed cSCC pathology report based on consensus recommendations.
