# Supplementary material for: Guidelines for Clinicians and Pathologists on Performing Skin Biopsies and Reporting on Suspected Cutaneous Squamous Cell Carcinoma
Source: Curr Oncol. 2025 Dec 4;32(12):689. doi: 10.3390/curroncol32120689 (PMC12732271; doi:10.3390/curroncol32120689)
Supplement: Supplementary file 1 [file curroncol-32-00689-s001.zip › curroncol-3934506-supplementary.pdf]

Back Matter

SUPPLEMENTARY MATERIALS

Figure S1: Proposed cSCC requisition form based on consensus recommendations.

Figure S2: Example of a body schema that can be helpful for the requesting clinician to communicate to the pathologist where skin biopsy samples have been taken.

Figure S3: Proposed cSCC pathology report based on consensus recommendations.

SUSPECTED cSCC PATHOLOGY REQUISITION FORM

PATIENT INFORMATION

Age: _________ Sex: _________

Immunosuppression status:

- ◯ Hematologic malignancy (e.g., leukemia, lymphoma)
- ◯ Organ transplant
- ◯ Other: ______________________________

Personal medical history: ______________________________

Previous procedures/therapies to the current lesion:

____

Other patient information

____

PATHOLOGY PROCESSING DIRECTIVES

Biopsy method used and purpose:

◯ Incisional (partial sampling of lesion):

- ◯ Punch
- ◯ Shave
- ◯ Saucerization
- ◯ Wedge

◯ Excisional (complete removal of lesion):

- ◯ Punch
- ◯ Shave
- ◯ Saucerization
- ◯ Ellipse

◯ Unknown

◯ Other (specify):

____

Re-excision ◯ No ◯ Yes (previous biopsy result: ___________________)

Expedited review

◯ No ◯ Yes (indicate planned procedure/next step):

- ◯ Radiation therapy
- ◯ Surgery
- ◯ Mohs
- ◯ Referral (specify): ______________________________
- ◯ Other (specify): ______________________________

Margin assessment:

◯ No ◯ Yes (indicate rationale): ______________________________

Other information: ______________________________

LESION INFORMATION

Lesion width/largest diameter: ______________________

New or recurrent lesion:

◯ New ◯ Recurrent

Chronic features:

◯ Wounds ◯ Ulcers ◯ Burns ◯ Other

Suspected nodal involvement:

◯ Yes ◯ No

Precise anatomic location of biopsy (body schema) and/or measurement from anatomic landmarks:

____

Clinical photos/images provided

◯ Yes ◯ No

Other lesion information: ______________________________

____

SUSPECTED cSCC BIOPSY LOCATION REPORT

Directions: Using the diagram, mark the biopsy location.

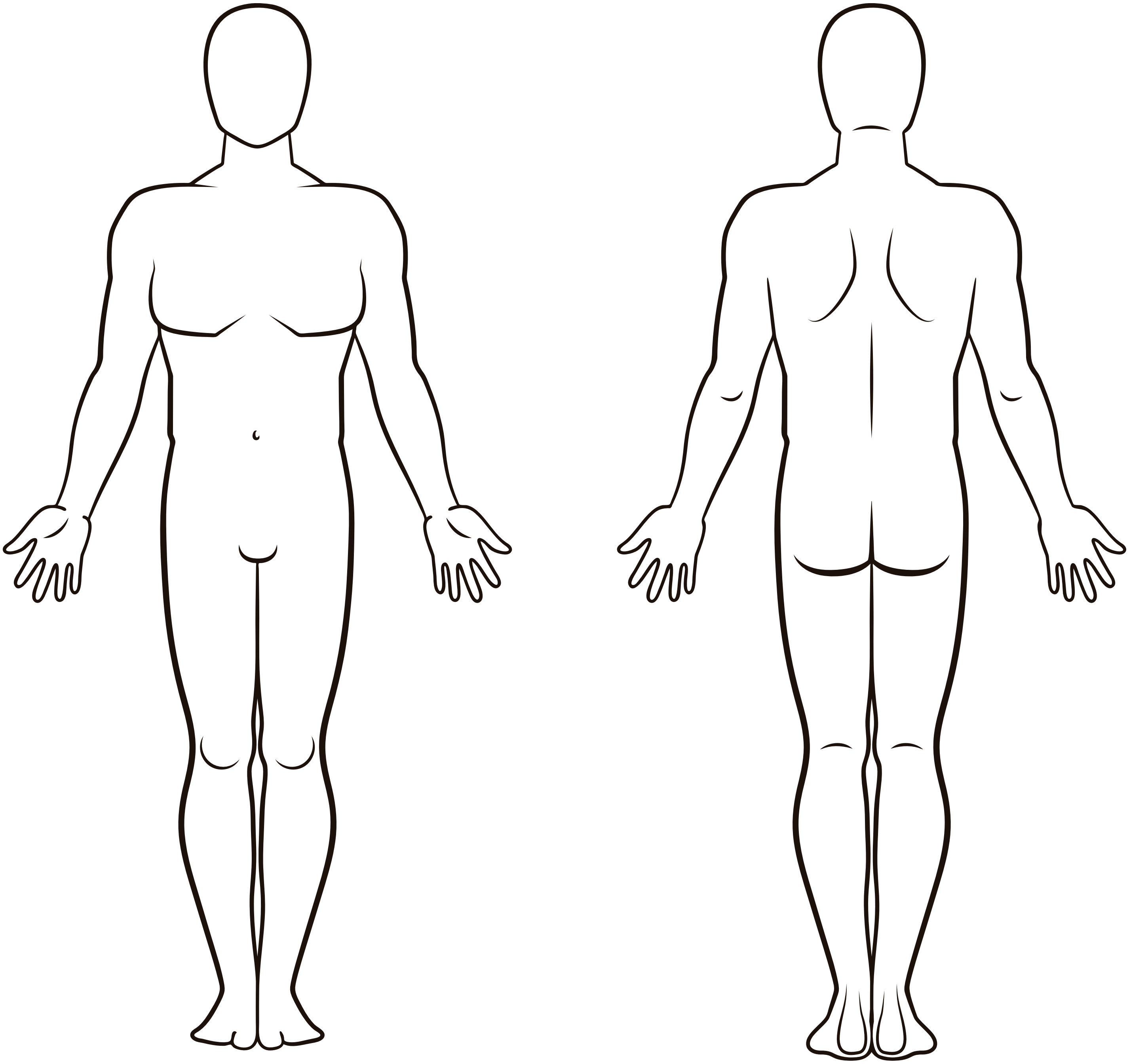

Provide a brief description regarding the lesion’s location with reference to precise anatomical landmarks:

___

___

___

SUSPECTED cSCC PATHOLOGY REPORTING FORM

HISTOLOGIC SUBTYPE

- ○ Classic SCC (NOS)
- ○ Keratoacanthoma
- ○ Acantholytic SCC
- ○ Spindle cell SCC
- ○ Verrucous SCC
- ○ Adenosquamous
- ○ Clear cell SCC
- ○ Other (specify): ______________________

DEGREE OF DIFFERENTIATION

- ○ Well differentiated
- ○ Moderately differentiated
- ○ Poorly differentiated
- ○ Undifferentiated
- ○ Cannot be assessed (reason): ______________________

LEVEL OF INVASION

- ○ Carcinoma in situ
- ○ Invasion of dermis and/or subcutaneous fat
- ○ Invasion beyond the subcutaneous tissue
- ○ Tumour with gross cortical bone/marrow, skull base invasion, and/or skull base foramen invasion

MAXIMUM TUMOUR THICKNESS

- ○ ______ mm
- ○ At least _____ mm
- ○ Cannot be assessed (reason): ______________________

PERINEURAL INVASION

- ○ Yes:
  - ○ Focal ○ Multifocal/extensive
  - Diameter of largest nerve involved: ○ <0.1 mm ○ ≥0.1 mm
- ○ No
- ○ Uncertain
- ○ Cannot be assessed (reason): ______________________

LYMPHOVASCULAR INVASION

- ○ Yes
- ○ No
- ○ Uncertain
- ○ Cannot be assessed (reason): ______________________

MARGIN STATUS ASSESSMENT

PERIPHERAL MARGINS:

- ○ Involved
- ○ In situ carcinoma
- ○ Invasive carcinoma
- ○ Not involved
- ○ Distance from margin: _____ mm
- ○ Uncertain
- ○ Cannot be assessed
- ○ Not applicable

Comment on location (if possible): ______________________

DEEP MARGINS:

- ○ Involved
- ○ In situ carcinoma
- ○ Invasive carcinoma
- ○ Not involved
- ○ Distance from margin: _____ mm
- ○ Uncertain
- ○ Cannot be assessed
- ○ Not applicable

Comment on location (if possible): ______________________

NODAL DISEASE

- ○ No lymph nodes submitted or found
- ○ Total number of lymph nodes examined: __________
- ○ Total number of lymph nodes involved: __________

Extranodal extension:
○ Not identified ○ Present

OTHER FACTORS TO COMMENT ON IF APPLICABLE

Aggressive Histologic Characteristics

Comment:

____

Number of high-risk features

Comment:

____

Additional comments

Comment:

____

cSCC: cutaneous squamous cell carcinoma; NOS: not otherwise specified; SCC: squamous cell carcinoma.
